# Screening for depression in elderly Indian population

**DOI:** 10.4103/0019-5545.64595

**Published:** 2010

**Authors:** Ankur Barua, Nilamadhab Kar

**Affiliations:** Department of Community Medicine, Kasturba Medical College, Manipal, Karnataka, India

**Keywords:** Cognitive impairment, depressive, disorders, prevalence, reliability, screening, validity, well-being

## Abstract

**Background:**

The point prevalence of depressive disorders in the elderly population in India varies from 13 to 25%. Since the World Health Organization (five) Well-being Index (1998 version) is simple and easy to administer, an attempt is made to evaluate the Indian version of this instrument to identify depression in the elderly Indian community.

**Objectives:**

(1) To determine the prevalence of depression among the elderly population of rural areas of Udupi district, Karnataka, India. (2) To determine the validity and reliability of WHO (five) Well-being Index (1998 version) as a screening instrument to identify depressive disorders in elderly population in this Indian setting.

**Materials and Methods:**

This cross-sectional study was conducted over a period of eight months (from March 1 to October 31, 2002) in the three taluks of Udupi, Kundapura, and Karkala; belonging to the Udupi district of South India. We selected 627 people in the age group of 60 years and above for the study. Simple random sampling, without replacement method, using the probability proportionate to size (PPS) technique was used. The WHO (five) well-being index (1998 version) was validated against the major International Classification of Diseases and Related Health Problems 10th Revision (ICD-10) depression inventory of mastering depression in primary care version 2.2. Proportions and their 95% confidence intervals were calculated and Kappa statistics was applied to determine the reliability of the screening instrument. *P* value<0.05 was considered statistically significant.

**Results:**

The prevalence of depression in elderly population was determined to be 21.7% (95% CI = 18.4 - 24.9). The Indian version of WHO-five well-being index (1998 version) showed a sensitivity of 97.0%, specificity of 86.4%, positive predictive value of 66.3% and an overall accuracy of 0.89. The Kappa statistics showed significantly high reliability of *k = 0.71*.

**Conclusion:**

The Indian version of "WHO (five) Well-being Index (1998 version)" was found to be an effective instrument for identifying depression in elderly Indian community.

## INTRODUCTION

The future projections of global disability adjusted life years (DALYs) in the year 2020 show that in terms of global disease burden, unipolar major depression could become the second leading cause in the disease burden after ischemic heart disease, especially in high-income countries.[1,2] A high prevalence of mental disorders is seen in old age. Predominant among these is *depression*. The Indian aged population is currently the second largest in the world.[1] Community-based mental health studies have revealed that the point prevalence of depressive disorders among the geriatric population in India varies between 13 and 25 %.[3,4]

Though depression is the commonest mental health problem in old age, very few community-based studies have been conducted in India to understand the problem. No similar study has been conducted in the past among the geriatric population in Udupi taluk of Karnataka state in South India and the WHO (five) Well-being Index (1998 version) was also never validated under an Indian setting before. Considering this background, we conducted a community-based mental health study in the rural area of Udupi taluk of Karnataka state in South India.

## MATERIALS AND METHODS

This cross-sectional study was conducted for eight months (from March 1 to October 31, 2002) in the three taluks of Udupi, Kundapura, and Karkala belonging to the Udupi district of South India. We selected 627 people in the age group of 60 years and above, who were permanent members of their respective households, for the study. Simple random sampling, without replacement method, using the probability proportionate to size (PPS) technique was used to select the respondents. Due to feasibility constraints and different on-going projects in other field practice areas of Kasturba Medical College, Manipal, only three villages were selected for this study.

### Sample size estimation

The sample size was estimated for finite population with the help of Expanded Position Indicator (EPI)-info version 5.0 for windows, statistical package. The total geriatric population (>= 60 years) in the three villages of Udayavara, Kadekar and Katapady was estimated to be 2259. Here, the confidence level was taken as 95%, prevalence rate of depression was 11.2%, required relative precision of the estimate was set at 20% and a non-response rate of 10% was included. Hence, the final sample size was determined as 627.

### Exclusion criteria

If a designated house was found locked during the first visit and the eligible residents could not be contacted, even after two successive revisits, they were all excluded from this study.

### Criteria to define non-respondent

If a designated respondent was non-cooperative or had severe behavioral problems or cognitive impairment; or had severe hearing impairment or articulation disorder, any terminal illness or; if he could not be contacted during two separate revisits after the first, he was considered a non-respondent.

### Study instruments

A face sheet consisting of information regarding the household of the respondent was used for data collection. A semi-structured proforma containing information regarding the socio-economic status of the individual that was later estimated by the modified Udai Pareek Scale[5] was also used. Presence of depressive disorders was determined using the instrument mastering depression in primary care, version 2.2: It had two components: (a) WHO (five) Well-being Index (1998 version), (b) Major (ICD-10) Depression Inventory. A previous study by Bonsignore M *et al*. conducted in Europe (Germany) to compare the validity of the first (1995 version) and the second (1998 version) of the WHO-(five) Well-being Index suggested that due to its higher Loevinger coefficient (0.38) and Mokken coefficient (> 0.3 in nearly all items), the second version (1998 version) was superior to the first version (1995 version) in detection of depressive disorders. The external validity ranked highly, as indicated by Receiver-operated-characteristic (ROC) analyses. WHO(five) scores were related to the absence or presence of depression.[6] In this study, the WHO (five) Well-being Index (1998 version) was validated against the Major (ICD-10) Depression Inventory, which was considered as gold standard by the WHO as it was based on the ICD-10 criteria.

Cognitive Impairment was estimated by the 6 -cognitive impairment test (CIT) dementia test. Mastering depression in primary care Version 2.2 and the 6-CIT dementia test were translated into Kannada and Hindi by the researchers and back-translated into English by another expert, who was not acquainted with the original versions. The back-translation was subsequently compared with the original version by a psychiatrist for conceptual equivalence of the items. The validity of 6-CIT at a cut-off of 7/8 is as follows: Sensitivity - 78.57%, Specificity - 100%, Positive Predictive Value - 100% and Negative Predictive Value - 83.33%.[7]

### Data collection procedure

The investigator, along with three field ANMs (auxiliary nurse mid-wives), were trained by the psychiatrists on how to administer the questionnaires. The chief investigator also accompanied the ANMs during their field visits and personally supervised the data collection procedure throughout this project. All the study instruments were pre-tested to determine whether they optimally suited the field conditions. After informed verbal consent was obtained, a brief general health check-up of the respondent was conducted to establish a good rapport with him. All the questionnaires administered in the field were evaluated and rated on the spot; if a respondent became positive in any of the screening or diagnostic instruments, he was immediately handed over a referral slip and sincerely requested to visit the Psychiatry Out-Patient Department (OPD) of Kasturba Hospital, Manipal of Karnataka, at the earliest, for a free consultancy. The participants with obvious medical disorders were referred to the nearest Rural Maternity and Child Welfare (RMCW) home run by Kasturba Medical College, Manipal of Karnataka, for a free health check-up. The diagnoses generated by the instruments in the study were kept strictly confidential and reconfirmed following consultations with a senior faculty member of the department of psychiatry of KMC Hospital, Manipal, before arriving at a final ICD-10 diagnosis for data analysis.

### Data analysis

The collected data were tabulated and analyzed using the statistical package Statistical Package for Social Sciences (SPSS) version 10.0 for Windows. Findings were described in terms of proportions and their 95% Confidence Intervals. Kappa statistics was applied to study the reliability of the screening instrument. *P* value less than 0.05 was considered as significant.

## RESULTS AND DISCUSSIONS

As part of the field survey, 487 households were visited and 627 individuals in the geriatric age group of 60 years and above were contacted. Among these 627 elderly people, we could interview only 609 for the assessment of depressive disorders (97.1%). Eighteen individuals, whom we could not interview due to various reasons, were categorized as non-respondents (2.9%). Majority (38.9%) of the non-respondents were unavailable at home during the first visit and could not be contacted later even after two successive home visits at a later date. Due to lack of practical skills in communication with the individuals suffering from severe hearing impairment and aphasia we were unable to interview them and they were also considered non-respondents.

The baseline characteristics of the population surveyed revealed that 36.0% were males while 64.0% were females. Majority (52.6%) of them belonged to the age group of (60-69) years. Only 58.7% of the elderly were literates and majority (61.2%) belonged to the middle socio-economic status. Most of the people were Hindus (80.1%) followed by Christians (13.0%) and Muslims (6.9%) respectively. Among the study population, 56.3% of the individuals were married while 43.7% were unmarried / widowed/ separated; none of them were divorced.

The overall prevalence of depressive disorders among the elderly of 60 years and above was found to be 21.7% (95% CI=18.4 - 24.9). The study findings were consistent with the observations made by Nandi DN *et al*[3] West Bengal, Ramachandran V *et al*.[4] and Tiwari SC,[8] who had determined the prevalence of depressive disorders in the elderly population to be 22.0%, 24.1% and 13.5% respectively. However, a high prevalence of depressive disorders of 52.2% among the elderly ≥ 60 years was observed in the study conducted by Nandi PS *et al*[9] in the rural areas of West Bengal.

It was found that the prevalence of significant cognitive impairment, suggestive of dementia, was higher among the depressed individuals (49.2%) as compared to the overall respondents (29.0%). The difference in prevalence of significant cognitive impairment among the depressed and not depressed individuals was found to be statistically significant (*x^2^* =11.522, *df* = 1, *P*>0.0001). This observation suggests that a proportion of depressed individuals were likely to suffer from concomitant dementia, which could not be identified in this cross-sectional study. Kay DW *et al*[10] reported about the concurrent co-existence of cognitive impairment and depressive symptoms in the community dwelling elderly. He had also observed that with cross-sectional data, it was not possible to comment on the changes that might take place in one in parallel with progression or remission in the other.

The status of positive well-being among the study subjects was also assessed by using the WHO (five) Well-being Index (version 1998). The prevalence of depressive disorders was high among individuals whose status of positive well-being was poor (75.9%) as compared to those whose status was satisfactory (5.3%).

As a follow-up of this study, nearly 90% of the individuals, who were tested to be positive by any of the screening instruments, were motivated and subjected to a thorough psychiatric evaluation at KMC Hospital, Manipal, and provided full treatment free of charge [Table 1, 2].

**Table 1:** Assessment of validity and reliability of WHO (five) well-being index (1998 version) as a screening instrument for identifying depressive disorders in elderly population in this Indian setting

**Validity:**	
Sensitivity: 97.0%	Specificity: 86.4%
Positive predictive value: 66.3%	Negative predictive value: 99.0%
False positive ratio: 0.14	False negative ratio: 0.03
Likelihood ratio (+ve Test): 6.9	Likelihood ratio (-ve Test): 0.03
Overall accuracy: 0.89	
**Reliability:**	
Kappa statistics: *k=0.71* and	
P=0.0001*	

**Table 2:** Validity and reliability of who-five well-being index (1998 version) as a screening instrument for identifying depressive disorders in elderly population in this Indian setting

Screening for depressive disorders in elderly population		Depressive disorders (Confirmation by Major (ICD-10) depression inventory along with clinical psychiatric assessment)		Total
		Present	Absent	
Who-five wellbeing index	Screening positive	128	65	193
	Screening negative	4	412	416
Total		132	477	609

**Figure 1:**
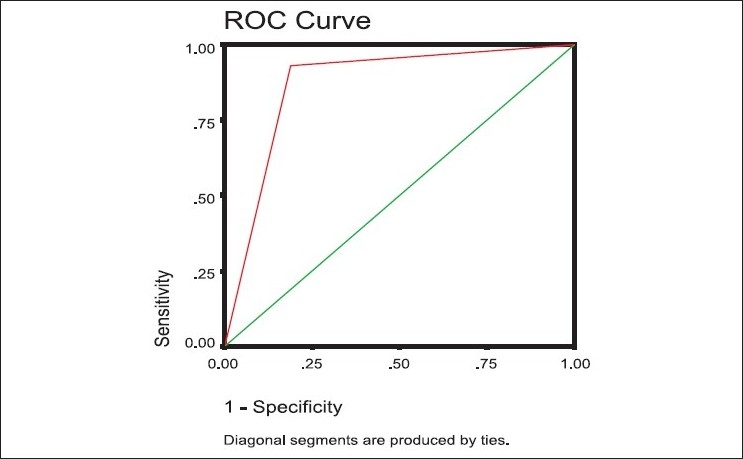
Assessment of external validity for detection of depressive disorders: evaluation through analysis of the roc curve

Figure 1 shows the assessment of external validity for detection of depressive disorders, an evaluation through analysis of the ROC curve for WHO-(five) Well-being Index (1998 version). The total area under the curve was 0.870 (SE=0.021, 95% CI=0.828-0.911, *P*=0.0001*). This was calculated under nonparametric assumption and for null hypothesis, where true area = 0.5.

Since, the WHO (five) Well-being Index (1998 version) showed a good internal and external validity and reliability, this is a useful instrument for identifying elderly subjects with depression in Indian community.

A previous study by Bonsignore M *et al*. showed that the WHO-5 has a good internal consistency and homogeneity, equivalent to the longer precursor versions of Well-being Index. This study was conducted in Europe (Germany) to compare the validity of the first (1995 version) and the second (1998 version) of the WHO (five) - Well-being Index.[6]

Due to its higher Loevinger coefficient (0.38) and Mokken coefficient (> 0.3 in nearly all items), the second version (1998 version) seemed superior to the first version (1995 version) for detection of depressive disorders. The external validity ranked highly, as indicated by ROC analyses. WHO (five) scores were related to the absence or presence of depression. These results suggest that the second version (1998 version) may be preferred in the future as a screening instrument for depression. The WHO (five) Well-being Index (1998 version) is a useful instrument for identifying elderly subjects with depression. The results are restricted to an elderly population at risk for psychiatric disorders; the transferability to other samples needs to be assessed in future.[6]

A study was conducted by Awata S *et al*,[11] to evaluate the validity and the utility of the Japanese version of the WHO (five) Well-being Index (WHO-five-J) in the context of detecting suicidal ideation in elderly community residents. In this study, the Cronbach’s alpha was 0.87 and Loevinger’s coefficient was 0.64. The internal validity showed sensitivity = 87%, specificity = 75%, negative predictive value = 99%, and positive predictive value = 10%.The receiver-operating characteristic curve analysis indicated that the scale significantly discriminated the subjects with suicidal ideation.

## CONCLUSIONS

In this study, the prevalence of depressive disorders among the geriatric population was determined to be 21.7% (95% CI = 18.4 - 24.9). The prevalence rates of depression among the males and females were 19.9% and 22.6% respectively. Since, the Indian version of WHO (five) Well-being Index (1998 version) showed a good Internal and external validity and reliability for identifying depressive disorders in elderly population, this could be considered a useful instrument for identifying elderly subjects with depression in Indian community.
